# Editorial: Molecular Insights Into Ligand-Receptor Interactions on the Cell Surface

**DOI:** 10.3389/fmolb.2022.921677

**Published:** 2022-05-13

**Authors:** Laura Marchetti, David Porciani, Stefania Mitola, Chiara Giacomelli

**Affiliations:** ^1^ Department of Pharmacy, University of Pisa, Pisa, Italy; ^2^ Department of Molecular Microbiology and Immunology, University of Missouri, Columbia, MO, United States; ^3^ Bond Life Sciences Center, University of Missouri, Columbia, MO, United States; ^4^ Department of Molecular and Translational Medicine, University of Brescia, Brescia, Italy

**Keywords:** ligand-receptor interactions, membrane receptor, CXCR4, receptor fluorolabeling, co-receptor

## Introduction

Interactions of endogenous or synthetic ligands with their target molecules on the plasma membrane modulate several biological processes, such as cell proliferation and differentiation. As a consequence, aberrant ligand-receptor interactions can promote several diseases, e.g., cancer or immunological disorders. Thus, most of the clinically-approved drugs target overexpressed and/or mutated cell-surface molecules, or their natural ligands (Bhullar et al., 2018; Insel et al., 2019). In addition, synthetic ligands with precise molecular recognition toward distinct receptors have been used for the targeted delivery of payloads into specific diseased cells (Mitchell et al., 2021). However, the study of how both endogenous and synthetic ligands affect the physiology and spatiotemporal regulation of their target receptors is challenging. This Research Topic provides an overview of multidisciplinary strategies used to study ligand-receptor interactions in different physio-pathological contexts.

Although the ligand-receptor interactions are pivotal to activate an intracellular signal transduction, interactions with additional membrane-associated molecules are often critical for modulating signal strength. The interaction of a cell-surface receptor with secondary effectors *in cis* (*i.e.*, other receptors or cytosolic proteins anchored to the cell surface) can facilitate receptor-ligand binding or can promote formation of multiprotein complexes that trigger precise signaling cascades. A classic example of this type of interactions is constituted by the Eph receptors (EphA and EphB receptor tyrosine kinases) and their ephrin ligands (ephrin-As and ephrin-Bs). Cecchini and Cornelison provided extensive insights on the molecular interactions of Eph receptors and how distinct interactions with their ephrin ligands and proximal proteins located *in cis* (on the same cell surface) can precisely modulate several cellular processes such as proliferation and differentiation, adhesion and migration, with emphasis on cancer development and progression, as well as cell sensing of the surrounding environment topography.

During the last decades, progress in structural biology has synergized with innovative computational methods to improve study of receptor dynamics and receptor-ligand interactions (Krishna Deepak et al., 2021). Wang et al. proposed a network pharmacology approach to explore the potential mechanism of *Tripterygium wilfordii* (TW) in the treatment of diabetic nephropathy (DN). The network pharmacology was first proposed by Hopkins et al. (Hopkins 2008) to overcome the so-called “one-drug/one-target/one-disease” approach in drug discovery. So far, several authors applied the network pharmacology approach mainly to elucidate the mechanism of action of several Chinese herbal medicine (Bi et al., 2021; Li et al., 2022; Wu et al., 2022). Wang et al. through a network analysis identified a series of potential targets of TW in the treatment of DN. The results were analyzed through GO biological process analysis and KEGG metabolic pathway and verified with molecular docking analysis. This approach allowed identification of a set of 49 targets involved in inflammatory process, oxidative stress insulin resistance, cell apoptosis and renal fibrosis, ultimately highlighting the specific molecular receptor involved in TW activity. Thus far, application of network pharmacology has been mainly applied for studies of Traditional Chinese Medicine. However, this approach and other computational methods have lately gained great attention in the field of ligand-receptor interactions and to study cell-to-cell communication. Indeed, over the past 5 years, computational methods and single-cell technologies have improved our knowledge of cell-specific ligand–receptor patterns in complex tissues, and provided a better understanding of cell communication (Armingol et al., 2021; Noël et al., 2021; Raredon et al., 2022).

The pipeline of drug development can be boosted by computational analysis, but a final validation with biological evidence is needed to move to pre-clinical and clinical settings. Several high-throughput screening (HTS) approaches have been proposed to improve studies of drug-receptor interactions (Yasi et al., 2020). Murphy et al. used molecular biology techniques to engineer *Saccharomyces cerevisiae* to express the GPCR CXCR4. The authors developed a tailored HTS approach that ultimately demonstrated the ability of the 2-carboxyphenyl phosphate (fosfosal) to bind and activate CXCR4 only in the presence of CXCL12.

Another important aspect of ligand-receptor interactions is to precisely define interaction dynamics. Radioligand binding assays, surface plasmon resonance analysis, and isothermal calorimetry are great tools to elucidate ligand binding, but they might not be sufficient to determine all receptor states, and often work in a cell-free system using recombinant proteins or peptide domains. On the other hand, atomic force microscopy and other high-resolution imaging techniques, such as transmission electron microscopy, only report static images (Lam et al., 2020). Single-molecule fluorescence imaging overcomes these limitations and can be used to investigate the dynamics and kinetics of different ligand-receptor interactions on live cells (Fernandes et al., 2021). Advanced fluorescence microscopy approaches have revolutionized the possibility to detect and quantify biomolecule complexes and receptors in dynamic cellular systems (Koldsø and Sansom, 2015; Sezgin, 2017). For instance, single-molecule imaging has defined how receptors are activated by their ligands, and how aberrant spatial distribution of membrane receptors is implicated in several pathologies, including nearly all types of cancer (Du and Lovly, 2018). With the advances of microscopy methods, new labeling approaches have emerged to enable precise coupling of fluorescently tags to cell-surface receptors (Wolf et al., 2021). Despite advancing the precision of labeling and visualization of membrane molecules, unfortunately certain fluorolabeling strategies can alter the native spatiotemporal regulation of the tagged receptors (Bosch et al., 2014; Hughes et al., 2014). Thus, the use of appropriate fluorolabeling strategies is pivotal to accurately elucidate receptor motion and localization on the cell surfaces in their native context, without altering physiological functions. In this respect, Amodeo et al. addressed receptor labeling limitations, with emphasis on fluorolabeling methods for live-cell imaging. This paper performed a systematic analysis to guide the chemical biology community in choosing proper fluorophores and methodologies to quantitatively define fluorolabeling efficiency and behavior of chemical tags coupled to membrane receptors in high sensitivity microscopy setups.

To date, several methodological approaches have been developed to better elucidate all aspects of ligand-receptor interactions. However, this research field is growing, and the limitations of different techniques will certainly prompt the development of new strategies that will improve our understanding of ligand-receptor interactions at the molecular level.
